# Pickering Emulsions Based in Inorganic Solid Particles: From Product Development to Food Applications

**DOI:** 10.3390/molecules28062504

**Published:** 2023-03-09

**Authors:** Andreia Ribeiro, José Carlos B. Lopes, Madalena M. Dias, Maria Filomena Barreiro

**Affiliations:** 1 Laboratory of Separation and Reaction Engineering—Laboratory of Catalysis and Materials (LSRE-LCM), Faculdade de Engenharia, Universidade do Porto, Rua Dr. Roberto Frias, 4200-465 Porto, Portugal; 2 Associate Laboratory in Chemical Engineering (ALiCE), Faculdade de Engenharia, Universidade do Porto, Rua Dr. Roberto Frias, 4200-465 Porto, Portugal; 3 Centro de Investigação de Montanha (CIMO), Instituto Politécnico de Bragança, Campus de Santa Apolónia, 5300-253 Bragança, Portugal; 4 SusTEC—Laboratório para a Sustentabilidade e Tecnologia em Regiões de Montanha, Instituto Politécnico de Bragança, Campus de Santa Apolónia, 5300-253 Bragança, Portugal

**Keywords:** Pickering emulsions, inorganic particles, productive technologies, food applications

## Abstract

Pickering emulsions (PEs) have attracted attention in different fields, such as food, pharmaceuticals and cosmetics, mainly due to their good physical stability. PEs are a promising strategy to develop functional products since the particles’ oil and water phases can act as carriers of active compounds, providing multiple combinations potentiating synergistic effects. Moreover, they can answer the sustainable and green chemistry issues arising from using conventional emulsifier-based systems. In this context, this review focuses on the applicability of safe inorganic solid particles as emulsion stabilisers, discussing the main stabilisation mechanisms of oil–water interfaces. In particular, it provides evidence for hydroxyapatite (HAp) particles as Pickering stabilisers, discussing the latest advances. The main technologies used to produce PEs are also presented. From an industrial perspective, an effort was made to list new productive technologies at the laboratory scale and discuss their feasibility for scale-up. Finally, the advantages and potential applications of PEs in the food industry are also described. Overall, this review gathers recent developments in the formulation, production and properties of food-grade PEs based on safe inorganic solid particles.

## 1. Introduction to Pickering Emulsions

Emulsions are considered one of the most important systems since they are widely used in many industries, including food, cosmetic, pharmaceutical and agrochemical sectors [1,2,3]. Emulsions are defined as a mixture of two immiscible liquids, where one liquid is typically dispersed in the form of droplets into another [1]. The phase corresponding to the droplets is called the dispersed phase, whereas the phase they are dispersed in is called the continuous phase [1,4]. Depending on the liquid forming the dispersed and continuous phases, two types of emulsions can be obtained: oil-in-water (O/W), consisting of oil droplets dispersed in a continuous aqueous phase; and water-in-oil (W/O), which corresponds to water droplets dispersed in a continuous oil phase [1]. More complex systems, i.e., double emulsions, can also be formed, namely the oil-in-water-in-oil (O/W/O) and the water-in-oil-in-water (W/O/W) types [1,5]. These emulsions are formed by dispersing the primary emulsion (O/W or W/O) into a continuous oil or water phase, respectively. For that, a second stabiliser should be used to guarantee emulsion stability [5].

Emulsions are formed by the intensive mixing of the two phases (water and oil). However, rapid phase separation occurs shortly after stopping the mixing due to the formed high interfacial forces. Thus, to produce an emulsion, the use of stabilisers (emulsifiers or surfactants) is mandatory [6]. Pickering emulsions (PEs) are stabilised by solid particles instead of conventional stabilisers, i.e., the emulsifiers or surfactants used in conventional emulsions (CEs) [7,8]. The ability of solid particles to physically stabilise emulsions was disclosed in the pioneering work of Ramsden [9] and Pickering [10]. Although the PEs concept emerged at the beginning of the twentieth century, interest in these systems has only risen in the last two decades, mainly in response to the constraints associated with the use of petroleum-related stabilisers, which have been associated with harmful health effects such as irritation and inflammatory responses [11,12].

**Figure 1:**
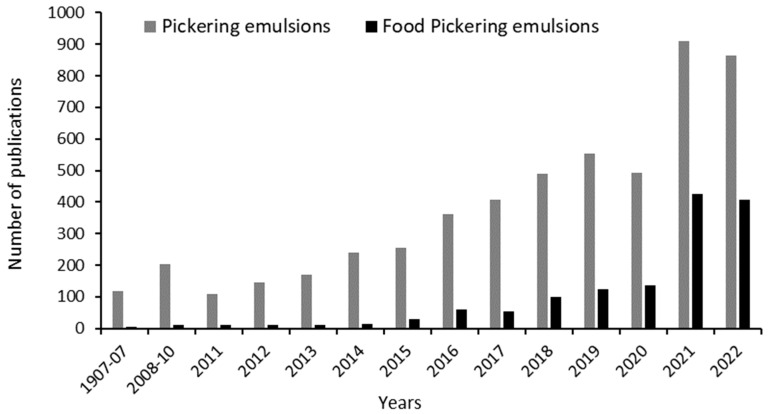
Number of publications in the last hundred-year period within PEs’ scope. The data were collected from the ISIweb of Science in February 2023 using the keywords PEs and Food PEs.

The search for solid particles compatible with food applications has gained an increased interest in the past few years, as evidenced by the large number of scientific publications in the field (Figure 1). PEs provide a way to develop functional emulsions with biocompatible solid particles [13,14] and enhanced chemical stability [15,16,17], enabling products with an emulsifier-free label [18]. In this context, PEs based on inorganic solid particles can be the basis of new alternative (e.g., vegan) functional and stable products, avoiding petroleum-based additives, and complying with cruelty-free practices, among others, responding to current lifestyle trends. Other applications have also increased, indicating PEs’ suitability for areas such as pharmaceuticals and cosmetics [19], oil recovery [3] and catalysis [20]. Thus, inorganic solid particles can have widespread use in emulsion stabilisation, conferring interesting characteristics on the final product.

Contrary to conventional stabilisers, the adsorption of solid particles at the interface is not spontaneous [21]. Solid particles should be insoluble in both dispersed and continuous phases, but with a preference for the continuous phase to improve emulsion stability, a fact related to its wettability (discussed in the Section 2*:* Mechanisms and Parameters Influencing Pickering Emulsion Stability) [22].

Currently, there are some inorganic particles used in PE stabilisation, such as silica or calcium carbonate. The main role of the solid particles in PEs is the formation of a physical and robust barrier at the oil–water interface preventing emulsion destabilisation, mainly against coalescence [23]. This barrier decreases the interfacial tension between two immiscible liquids, as happens with a conventional stabiliser [24].

Hydroxyapatite (HAp) is a biocompatible material with the potential to be used as a Pickering stabiliser [25], and is an interesting material for food application development. In this scope, some authors reported the production of PEs stabilised with HAp combined with polymeric materials to improve emulsion stability [26,27]. Recently, stable PEs using only HAp particles have been produced [25]. However, these studies are still under development, and are important to assembling the existing literature information on the topic.

This review aims to provide an updated overview of PEs stabilised with inorganic solid particles, systems that are attracting increased interest in the scientific community, opening new avenues for the development of innovative products. Thus, it is important to understand how stabilisation and functionalisation are guaranteed through these particles. In this context, the following topics will be addressed: particle characteristics (wettability, size, shape and surface charge), types of solid particles used, methods for PE development and production, and PE application in the food industry. A special focus will be directed to hydroxyapatite particles as Pickering stabilisers, regardless of their size. Overall, this review will provide the latest progresses and insights concerning the field of PEs.

## 2. Mechanisms and Parameters Influencing Pickering Emulsion Stability

### 2.1. Pickering Emulsion Formation Mechanism

Several works reported the mechanisms governing PE formation and respective stabilisation [2,6,28,29]. For PEs, the stabilisation mechanism is based on particle adsorption at the oil–water interface, resulting in a reduction of the interfacial area, conferring distinctive physical and mechanical properties compared to CEs [2,7]. The physical stability of the PEs is achieved through different mechanisms, including (1) capillary forces, (2) particle–particle networks, and (3) desorption energy.

Capillary forces refer to attractive interactions among the solid particles at the oil–water interface. Usually, the overlap of interfacial perturbations of two close particles causes an attraction, which induces the formation of a strong interfacial shell [24,30]. The capillary forces depend on wettability, particle size [24] and particle shape’ namely, non-spherical particles can improve the perturbations, resulting in increased forces [31].

Particle–particle networks address the attraction interactions among solid particles remaining in the continuous phase and/or particles covering droplets, forming unique and flocculated network structures [30]. The network is enhanced due to particle bridging—which occurs when a particle is adsorbed onto a droplet and interacts with a neighbour droplet interface [24]—and particle aggregation, i.e., when the particles aggregate, creating a disordered network stabilising, simultaneously, all the particles [24]. These particle–particle networks can improve the emulsion stability since this tight network prevents the coalescence of the dispersed phase [24].

Desorption energy pertains to the interactions between solid particles and the dispersed and continuous phases. The desorption energy is related to the free energy involved in removing an adsorbed particle from the oil–water interface [30]. The main characteristic of PEs is the high energy required to remove the particles adsorbed at the interface. The amount of energy needed to remove a spherical particle from the interface, i.e., the desorption energy, can be expressed by Equation (1) [32].

(1) $$∆G_{d}=\pi r^{2}\gamma_{ow}{\left(1-\left|\cos\theta \right|\right)}^{2}$$

where r is the radius of the solid particle, $\gamma_{ow}$ is the oil–water interfacial tension and θ is the contact angle. The desorption energy depends on the contact angle, particle size and interfacial tension (Equation (1)). The energy required for the desorption of solid particles with appreciable wettability is higher than the thermal energy, and hence the particles are considered as irreversibly adsorbed at the interface [2,24].

The main mechanism by which solid particles stabilise PEs relates to their interaction energy with the oil–water interface. Thus, particle desorption energy is the main factor that, theoretically, indicates how well particles with different characteristics stabilise O/W, W/O or double emulsions [24,30,33,34].

### 2.2. Pickering Emulsion Parameters—Particle Properties

The effectiveness of solid particles as stabilisers depends mainly on their wettability. However, factors such as particle size, shape and concentration, electrolyte concentration, pH of the continuous phase, oil type and volume fraction must be also considered [35]. Thus, these parameters and their role in emulsion stabilisation are revised in the next subsections.

#### 2.2.1. Particle Wettability

When particles are used to stabilise emulsions, wettability can be assumed as a parameter equivalent to the hydrophilic–lipophilic balance (HLB) used in CEs, since both concepts are related to the affinity of the particles or the surfactant/emulsifier to the oil and water phases [36]. Particle adsorption at the oil–water interface is strongly influenced by wettability, which is, in turn, related to the hydrophobicity of particles [24].

Solid particle wettability is characterised by the three-phase contact angle (θ) [7,28,32], which results from the balance of the interfacial tensions at the water–oil, particle–water and particle–oil interfaces, expressed by Young’s equation (Equation (2)).

(2) $$\cos\theta =\frac{\left(\gamma_{po}-\gamma_{pw}\right)}{\gamma_{ow}}$$

where, $\gamma_{po}$, $\gamma_{pw}$ and $\gamma_{ow}$ are the interfacial tensions of particle–oil, particle–water and oil–water interfaces, respectively.

**Figure 2:**
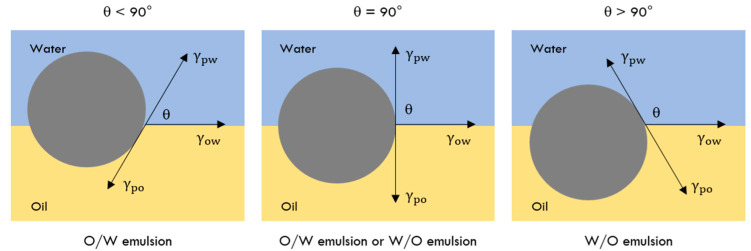
Influence of contact angle, θ, on the location of a solid particle at the oil–water interface. Adapted from [24].

The emulsion type follows the empirical Finkle rule admitting that θ is directly linked to the type of stabilised emulsion, O/W or W/O (Figure 2) [37]. Thus, for mono-layered solid particle stabilisation, with a contact angle ranging from 15° < θ < 90°, the particles are preferentially wetted by water (hydrophilic characteristics) and therefore suitable for forming O/W emulsions, whereas when the contact angle is between 90° < θ < 165°, the particles are preferentially wetted by oil (hydrophobic characteristics), being suitable for forming W/O emulsions [22]. In some cases, the solid particles have identical affinity for both water and oil phases, θ = 90°, and, theoretically, can stabilise O/W or W/O emulsions [2,7,35,38]. However, when a multi-layer of particles stabilises the droplets, the contact angle may change slightly; in this case, for 15° < θ < 129° O/W formation is expected and for 51° <  θ < 165° W/O formation is favoured [22].

Particle wettability is important to be considered since it determines the emulsion type, ensuring the appropriate anchoring of the solid particles at the interface as well as their efficiency in the stabilisation process [2]. However, the solid particles can be surface modified to change the contact angle and improve their original wettability. For example, for an O/W emulsion, enhancement of the particle/oil affinity decreases the initial repulsive force due to the decrease in the repulsive hydration force [39]. In this case, the particle/droplet contact is favoured, being an expected improvement in emulsion stability. Furthermore, increasing particle/oil affinity by surface modification also promotes the attachment of the particles at the droplet surface through capillary forces as a consequence of the improved wettability [39].

Particle modifications are performed to enable better anchoring at the interface, controlling emulsion type and stability [38,40,41]. Various authors worked on the development of Pickering stabilisers through surface modification [38,41,42,43,44,45]. These studies contemplate both inorganic and organic particles through physical or chemical methods using small molecules or polymers [38,46]. Palmitic and oleic acids were added to the emulsion system to modify silica particles, improving their adhesion at the droplet surface, and increasing the stability at four and three months, respectively [42,43]. In work developed by Björkegren et al. [44], the silica surface was modified with hydrophilic (methyl poly(ethylene glycol) silane) or hydrophobic compounds (organosilanes such as ethoxy trimethylsilane, dimethoxy dimethylsilane, triethoxy propyl silane, trimethoxy propyl silane and triethoxy octyl silane), with O/W emulsion stability strongly depending on the amounts of these compounds used. Additionally, the authors reported that the emulsification performance was further improved by the combination of both hydrophilic and hydrophobic groups; this heterogeneous modification led to emulsions with high stability towards coalescence (from five weeks to 1.5 years) [44]. CaCO_3_ particles were modified by fatty acids (sodium carboxylates), resulting in the formation of O/W or W/O emulsions [47]. The only carboxylate not giving rise to phase inversion was C6Na. For all the other studied carboxylates (C8Na, C10Na and C12Na), an improvement in O/W stability and phase inversion from O/W to W/O was observed. The required concentration for phase inversion decreased with chain length increase [47]. HAp particles were modified with sodium oleate by Ribeiro et al. [48]. The modified HAp particles presented a wettability dependent on the SO content used, switching from hydrophilic (initial state) to hydrophobic (one SO layer) and back to hydrophilic (two SO layers).

Feng and Lee [45] modified zein particles with low wettability to improve PE stability with sodium caseinate (NaCas) via ultrasound treatment to form zein/NaCas colloidal nanocomplexes (used zein/NaCas ratios: 10:1 to 10:4 at pH 3). The PEs produced from the zein/NaCas particles exhibited greater centrifugal stability than those using pristine zein particles. Another material commonly modified is starch since the native granules are not suitable for creating stable PEs [49]. In this case, the hydrophobicity is increased through esterification with octenyl succinic anhydride (OSA), acetic anhydride or phthalic anhydride or by heat treatments [13,34,46,50,51,52,53,54,55]. For example, although starch is a natural material with Generally Recognized as Safe (GRAS) recognition, the degree of substitution by OSA cannot exceed 3% (the maximum amount recommended by the FDA) in food-related applications [46]. These treatments are mostly applied to achieve the desired particle wettability; however, problems may arise since non-food grade solvents are used, implying possible particle contamination.

#### 2.2.2. Solid Particle Concentration

A direct relationship between particle concentration and emulsion stability has been reported [16,56], where the increase in solid particle concentration results in improved PE stability over time. The availability of more particles promotes the formation of a tighter-packed layer around the emulsion droplets [16]. Hence, coalescence (the main destabilisation mechanism) is prevented for a long period of time [32]. Furthermore, the increase in solid particle concentration can result in a droplet size reduction, which is also a stability-promoting factor [28].

Frelichowska et al. [56] observed a change from an unstable to a stable emulsion with an increased concentration of silica particles from 1 wt% to 9 wt%. For higher particle concentrations, the emulsion was stable over 2 years. Furthermore, the authors compared emulsion droplet size as a function of silica content increase, where a reduction from 15 µm to 1.5 µm was obtained when using the lowest and highest silica concentrations, respectively. Regarding HAp particles, PE stability was improved with a particle concentration increase, giving rise to stable emulsions (2-month period) for concentration above 5 wt% [25]. Kargar et al. [16] found that when microcrystalline cellulose or modified starch concentration increased from 0.1% to 2.5%, the size of the droplets decreased, and the physical stability of a sunflower O/W emulsion was enhanced, revealing stability for 40 days. The authors report that the increase in the solid particle concentration can be associated with PE stability improvement, mainly against coalescence. Considering the reported stability values for inorganic and organic solid particles, it is possible to observe that inorganic particles enable better performance. This is an important feature for the development of stable products.

#### 2.2.3. Particle Size

Particle size also affects emulsion formation and stability [24]. A relationship between particle and droplet sizes has been reported, assuming that particles should be substantially smaller than emulsion droplets [2,35,57]. Gould et al. [58] found that solid particles should be at least one order of magnitude smaller than emulsion droplets. This difference is recommended for enabling the formation of a structured interface layer around the droplets and for improving adsorption energy (see Equation (1)), which is proportional to the contact area [24,58].

Currently, the particles used to stabilise PEs tend to be in the nanometric size range. For inorganic solid particles, the smallest used size is around 5–10 nm [42] and the highest around 800 nm [59]. For example, Köhler et al. [60] studied the effect of using different silica sizes in PE stabilisation. It was observed that a reduction in particle size from 200 to 12 nm decreased droplet size from ~4 µm to ~14 µm. Additionally, compared to the use of Tween 20, the 12 nm silica particles demonstrated an ability to stabilise droplets within the same size range [60]. Considering the literature information, it is noticeable that larger inorganic particles, in the order of micrometres, are not typically used for PE stabilisation.

#### 2.2.4. Particle Shape

**Figure 3:**
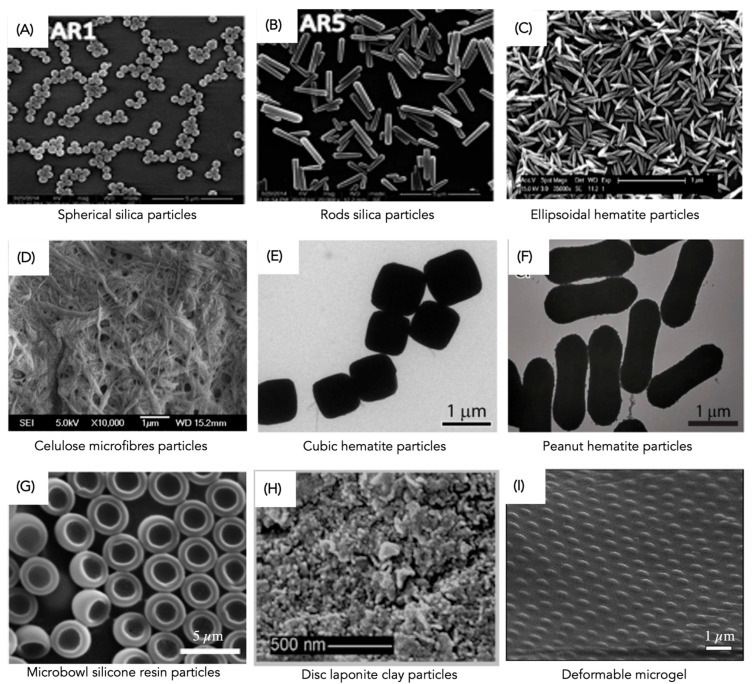
Different solid particle shapes. (**A**) spherical [31], (**B**) rods [31], (**C**) ellipsoidal [61], (**D**) microfibres [62], (**E**) cubic [63], (**F**) peanut [63], (**G**) microbowl [64], (**H**) disc [65] and (**I**) deformable gels [66]. (Reprinted from [31], Copyright (2016), with permission from the Royal Society of Chemistry; Reprinted from [61], Copyright (2009), with permission from the Royal Society of Chemistry; Reprinted from [63], Copyright (2014), with permission from the American Chemical Society; Reprinted from [64], Copyright (2011), with permission from the American Chemical Society; Reprinted from [65], Copyright (2009), with permission from the Royal Society of Chemistry; Reprinted from [66], Copyright (2009), with permission from the Royal Society of Chemistry).

Considering particle shape, stabilisation performance was mostly studied for spherical particles and many models were developed based on this morphology, e.g., desorption energy (Equation (1)) [24]. However, most solid particles used are not spheric, resulting also in effective solutions for the stabilisation of O/W or W/O PEs. For example, rod-shaped [31], ellipsoidal [61], fibre-like [62], cubic [63], peanut-shaped [63], microbowl-like [64], disc [65] and deformable gel [66] are among the reported typical non-spherical particles (Figure 3). In the food industry, particle shape is an important factor since food-grade or food-compatible particles usually have an irregular or anisotropic shape; thus, their impact on PE stabilisation is worthy of assessment [61].

Lou et al. [31] reported that O/W PE stability strongly depends on the silica rods’ aspect ratio. The emulsions stabilised with silica rods are stable for a longer period (a few months) than spherical silica with similar sizes (a few hours) [31]. The emulsion stabilisation was improved for higher aspect ratios, which was attributed to higher steric hindrance, interfacial adsorption energy and capillary forces. Similar results were obtained by Madivala et al. [61] with ellipsoidal hematite particles. Folter et al. [63] reported good emulsion stability against coalescence, up to a one-year period, using hematite cubic- or peanut-shaped particles. These particles revealed unique interfacial packing ability and orientation, improving the irreversible attachment of the particles to the droplet surface. Specifically, cubes were attached at the interface in monolayers oriented parallel to one of their flat sides, while the peanut-shape particles were attached as interdigitating stacks and oriented with their long axes parallel to the interface [63]. Lou et al. [31] reported in their work that the PE stability is improved when silica rod-like particles are used instead of their spherical counterparts, achieving longer stability (a period of months for rod-like particles against a few hours for spherical particles). Furthermore, PE stability can be enhanced using disc-type particles [67,68] and deformable materials [66]; these can adapt to the droplet surface, inducing efficient coverage, and preventing creaming and coalescence. Creighton et al. [67] stabilised PEs using ultrathin plate graphene oxide particles (similar to a disc shape), showing that this material has potential for emulsion stabilisation for controlled released applications since they significantly impact the dispersed phase evaporation. Additionally, the authors developed a thermodynamic model to predict the effect of material surface chemistry and geometry on PE stability. The model successfully predicts that graphene oxide, but not pristine graphene, has a favourable hydrophobic/hydrophilic balance for O/W emulsion stabilisation.

Although the stabilisation mechanisms of non-spherical solid particles are not yet fully elucidated, it is recognised that such particles can improve emulsion stability. Non-spherical particles can contribute to achieve better interfacial coverage, resulting in unique interfacial network properties [2]. When anisotropic particles, such as rods and ellipsoids, are used, there is enhanced network formation among the particles positioned at the droplets’ surface, resulting in stable PEs over time [68]. Thus, it is important to depict shape anisotropy effects on interfacial particle packing and orientation, as well as on capillary interactions [24].

### 2.3. Pickering Emulsion Parameters—Aqueous Phase Properties

Control of electrolyte concentration and pH of the continuous phase is often required to guarantee a balanced repulsion among particles and droplets [69]. The pH of the system alters the particles’ surface charge, affecting electrostatic interaction [69]. Thus, changes in the continuous phase can lead to a decrease, or even suppression, of the electrostatic repulsion between the solid particles and the oil–water interface, modifying particle–particle interactions and leading to a change from repulsive to attractive forces [28,69]. This can induce the aggregation of the particles into flocs, which, in turn, influence emulsion stability [2,32]. 

The effect of electrolyte concentration and pH have been studied in some Pickering particles, such as hydroxyapatite [70], clay [71], hydroxide particles [72] and other organic particles [73]. Various authors have reported that the presence of electrolytes can be a positive factor in achieving stable PEs [71,72,73]. Partial flocculation, i.e., a moderately prevalent attraction between particles, seems to provide better particle adsorption at the interface, improving PE stability [32,74]. Ribeiro et al. [70] studied the effect of pH and ionic strength in PEs stabilised with HAp particles. In general, the HAp PEs were stable within the tested ionic strength range (100–500 mM) and in relatively high pH environments (6–10); however, PEs undergo complete phase separation at very low pH (2) due to n-HAp particle disruption.

### 2.4. Pickering Emulsion Parameters—Oil Phase Properties

Oil type and the oil/water ratio can influence emulsion stability and the formed emulsion type (O/W or W/O) [28].

Oil type—which can range from non-polar hydrocarbons, with relatively high interfacial tension ($\gamma_{OW}$, e.g., heptane (50.7 mN/m) and dodecane (52.5 mN/m) [75]), to polar alcohols and esters with relatively low $\gamma_{OW}$ (e.g., eugenol (9 mN/m) and undecanol (9.5 mN/m) [75])—has an important role since it determines the interfacial tension at the oil–water interface, influencing interactions with solid particles (three-phase contact angle) [28]. Oil properties, such as polarity and viscosity, have a substantial impact since they directly affect the θ value [2,28,32]. Binks and Lumsdon [75] studied the effect of different oils using silica particles with intermediate hydrophobicity. They found that the emulsion type is O/W for non-polar oils and W/O for polar ones. Bai et al. [76] studied the influence of the oil type on PE stabilisation using cellulose nanocrystals, choosing different oils such as corn, fish, sunflower, medium-chain triglycerides, flaxseed and orange. They reported stable PEs for all oil types at 0.75 wt% cellulose nanocrystal content. After 14 days, all emulsions appeared to have good stability against coalescence, except the ones prepared using orange oil. For this formulation, an increase in droplet size was observed due to Ostwald ripening destabilisation. This observation was associated with orange oil’s high polarity and water solubility compared to medium- and long-chain triglyceride oils [76].

The oil viscosity influences the efficiency of oil breakage to form droplets during the emulsification process. Tsabet and Fradette [39] stabilised emulsions with glass beads, reporting a droplet size increase of 36% when silicone oil viscosity increases above 486 mPa·s. For higher oil viscosities, the authors reported that the emulsification process and interfacial adsorption ability of the particles were affected. In terms of stabilisation, higher oil viscosity retards particle adsorption at the oil–water interface, promoting coalescence phenomena. Stable PEs were produced with oil viscosities between 9.35 and 194 mPa·s. 

Emulsion type and stability are greatly influenced by the dispersed phase volume fraction [28]. Emulsion stability can be improved by increasing the oil phase (up to a limit) since this can increase the viscosity of the system, retarding phase separation [1]. However, when the oil volume fraction exceeds this limit, emulsion phase inversion is favoured. Binks and Lumsdon [77] report the formation of water-in-toluene PEs stabilised with either hydrophobic or hydrophilic silica particles. The authors found that PEs stabilised with hydrophobic silica can be inverted from W/O to O/W type upon increasing the water volume fraction. Inversely, PEs stabilised with hydrophilic silica can be inverted from O/W to W/O type upon increasing the oil volume fraction. The point where the catastrophic inversion occurs was similar for both systems, at around 0.7 (volume fraction of the dispersed phase). Additionally, Binks and Lumsdon [75] studied the water volume fraction needed—at least 95%—for phase inversion using different oil types (heptane, dodecane, toluene, isopropyl myristate, methyl myristate cineole, undecanol and eugenol) and silica as solid particles.

## 3. Inorganic Solid Particles as Pickering Stabilisers

Solid particles must present a set of characteristics to govern or control PE stabilisation. In this context, this section describes the main solid particles used as Pickering stabilisers. The principal focus will be food-grade particles, and special attention will be given to hydroxyapatite particles.

### 3.1. Types of Inorganic Solid Particles

The available types of solid particles able to be adsorbed at liquid interfaces and stabilise emulsions tend to grow. The solid particles commonly used can be divided into safe inorganic particles and food-grade organic particles [7]. Within organic particles, polysaccharides [7], proteins [24] and lipids [78,79,80] are mainly used. More information on PEs using organic particles can be found elsewhere [2,81].

Within the safe inorganic particles group, silica (SiO_2_) particles are the most studied since their surface can be modified through chemical or thermal treatments in order to change properties, mainly wettability [82]. Silica particles have been previously reported as food-grade particles [57,83,84]. EFSA—European Parliament and Council Directive No. 95/2/EC approved the use of silica and silicates (E551–E559) as food additives; even in some food products, their use is limited to a maximum content (e.g., 10 g/kg in powder foods) [85]. In contrast, dietary food supplements and foodstuffs in the form of tablets with or without silicate coatings can be used in “quantum satis” [85,86]. In addition, calcium carbonate (E170) is a safe inorganic particle used as a Pickering stabiliser [87], and is authorised as a food additive by EFSA—Commission Regulation 1129/2011 [88].

The ability of different materials to be used in the preparation of new particles has been investigated, and the potential safety risks of these materials need to be examined [24]. Their use in the food industry still involves some scepticism because assertive legislation for their safe application is needed [24]. Nevertheless, several PEs have been developed and studied to investigate and understand the behaviour of particles in PE stabilisation. Only in this way is the collection of essential data to make a cautious and scientifically correct decision possible. Table 1 summarises previous work using safe inorganic food-grade Pickering particles, showing, when data are available, the type, shape and size of the solid particles, parameters influencing PE stabilisation, aqueous and oil phases used, as well as the applied production method.

From the information gathered in Table 1, it can be seen that the most common PEs are of the O/W type, with fewer works dealing with W/O emulsions. In some cases, the solid particle’s surface was modified, targeting better stability performance, but also W/O emulsion production. From the perspective of developing food-grade emulsions, and considering the analysed data, it should be mentioned that many reported processes use non-food compounds. Some emulsions contain non-food-grade oil phases, such as toluene, hexane or silicone oil, non-food-grade emulsifiers such as sodium dodecyl sulfate (SDS), and non-food-grade modifier agents such as cetyltrimethylammonium bromide (CTAB). Hence, although the studies have been carried out with food-grade solid particles, further work must be done to develop more realistic systems for food purposes.

W/O emulsions were produced after surface modification using, e.g., molecules such as fatty acids. Solid lipid particles are one of the few particles that stabilise W/O emulsions without previous surface modification [23]. Thus, lipid molecules can help to tailor the solid particle wettability. Calcium carbonate was also modified with fatty acids to promote longer stabilisation times of O/W PEs [47,109]. The authors produced unstable emulsions when using only the carbonate particles, and emulsions stable for up to several months when adding fatty acids at a concentration of up to 60 mM [47].

Eskandar et al. [90] studied the synergistic effect of using silica particles combined with lecithin or oleylamine on the formation and long-term stability of O/W PEs. The work reported that when silica particles are initially added to the oil phase, an improvement in emulsification and stability against coalescence was observed (three months), a fact related to the synergistic effect generated between the particles and lecithin or oleylamine. Hydrophilic silica particles were modified with sodium caseinate, Tween 20, Tween 60 and different fatty acids targeting the stabilisation of O/W PEs [42,43,44,83,91,92,93,100,101,104].

Skelhon et al. [84] stabilised water-in-sunflower-oil emulsions using fumed silica particles treated with chitosan, which becomes surface-adsorbed. The authors reported that the combination of fumed silica particles and chitosan under acidic conditions (pH 3.2–3.8) was highly beneficial for the enhancement of emulsion properties in terms of dispersed phase volume and emulsion stability, compared to emulsions prepared using these compounds individually [84]. The combination of silica and chitosan promoted the adsorption of the silica particles at the droplet interface, resulting in stable emulsions for 24 h [84]. Björkegren et al. [44] used silica particles covalently modified with methyl poly(ethylene glycol) (hydrophilic character) or organosilanes containing propyl and methyl groups (hydrophobic character) trying to mimic surfactant properties to stabilise W/O emulsions. The authors reported that colloidal silica functionalized with the hydrophobic groups produced emulsions with smaller droplets (~10 µm), compared to the ones using unmodified silica (~18 µm). The emulsification performance was improved by combining hydrophilic and hydrophobic groups, generating stable emulsions against coalescence (from 5 weeks to 1.5 years). Cui et al. [47] controlled the emulsion type (O/W or W/O) by the amount and type of fatty acids, according to chain length, C6Na, C8Na, C10Na and C12Na, adsorbed at the calcium carbonate surface. The emulsion type depended on the chain length and fatty acid concentration; namely, the required concentration decreased as the chain length increased. For C6Na, emulsion inversion was verified with 60 mM, while for C12Na, the inversion occurred at concentrations below 3 mM.

Recently, Pickering stabilisers have also been applied to produce double emulsions. In the food industry, these emulsions can provide additional protection and controlled release of bioactive compounds and mask the flavour or unpleasant taste of some nutritional components [110]. Additionally, these emulsions can reduce fat content in processed food products [110]. However, these systems are still challenging due to the different natures of the needed stabilisers (hydrophilic and lipophilic). Different authors have worked on double PEs, but in some cases, only one emulsion was stabilised with a Pickering stabiliser. Examples include polyglycerol polyricinoleate (PgPr) to stabilise the primary emulsion and solid particles to stabilise the secondary one [34,111,112,113]. In the work of Zou et al. [105] the primary W/O emulsion was stabilised using hydrophobic particles (silica and oleic acid-coated Fe_2_O_3_), and the double emulsion (W/O/W) was stabilised with hydrophilic particles (clay, Fe_2_O_3_ or microgel). O/W/O emulsions were also obtained where the primary O/W emulsion was stabilised by hydrophilic particles and the double emulsion by hydrophobic ones [105].

The main concern in producing emulsions, particularly PEs, is their stability, along with storage time. Their integrity over time is essential to ensure, in the case of the food industry, product quality during shelf-life. Hence, most of the work shown in Table 1 was focused on checking emulsion stability over time. However, parameters such as particle size and concentration, aqueous or oil phase volume fraction and pH should also be considered and adjusted to ensure a product with long stability. 

Recently, PEs have also attracted attention when acting as systems to avoid oil oxidation (when oil is in a dispersed phase) or to provide the encapsulation of various compounds, such as vitamin E [114], vitamin D [115], carotenoids (β-carotene) [116] and curcumin [117]. The development of stable emulsions, both physically and chemically, enabled the obtaining of enhanced, or even new, approaches to active compound delivery. Kargar et al. [95] studied lipid oxidation using O/W PEs stabilised with silica particles, comparing their performance with CEs stabilised with sodium caseinate and Tween 20. They reported that emulsions stabilised by silica particles reduced, by around 50%, the oxidation rate when compared with the use of a conventional emulsifier. Tikekar et al. [99] encapsulated curcumin using silica particles to stabilise canola oil/water emulsions. The results showed that silica-stabilised PEs can maintain the stability of the encapsulated curcumin, which was approximately 100-fold higher than the curcumin suspended in water.

Hydroxyapatite is widely used in biotechnological applications due to its excellent biocompatibility [118]. Recently, hydroxyapatite particles have been studied as Pickering stabilisers to achieve emulsifier/surfactant-free emulsions, which could be the basis of innovative product development [114]. Thus, it is relevant to summarise the data available in the literature and to provide a critical evaluation of the use of HAp particles for PEs.

### 3.2. Hydroxyapatite as Pickering Stabiliser

Calcium phosphates are well-established biomaterials and have been used in biomedical products [119], cosmetics [120] and toothpastes [121]. They are common minerals encountered in nature being produced in different environments, such as geological (igneous apatite), geochemical (phosphorite) and biological (biological apatite) [122].

Hydroxyapatite (HAp) is a double salt of tricalcium phosphate and calcium hydroxide with a stoichiometric formula Ca_10_(PO_4_)_6_(OH)_2_. HAp has a Ca/P ratio of 1.67. Different Ca/P ratios can lead to phase impurities originating in other calcium phosphates [123,124]. HAp is poorly soluble, or even almost insoluble, in basic and neutral pH solutions, but soluble in acid solutions, typically at pH ≤ 4 [125]; however, the solubility depends on factors such as particle shape, size, porosity and crystallinity [124]. HAp is used as bone substitution material [126] and in various kinds of toothpaste to enhance tooth repair [121]. These applications are due to good biocompatibility, absorbability and osteoinductivity [125]. Recently, HAp has been described as a promising material for PE stabilisation [127]. In the food sector, using particles instead of conventional emulsifiers presents advantages in terms of physical stability and improvement of the functionality and performance of food emulsions [7], adding the possibility of producing clean-label products.

HAp and calcium phosphates are also used as calcium sources—an essential element for all biological organisms—in food supplements [128]. Calcium phosphates received the GRAS statement from the FDA, and their use in food was recognised as safe by the same institution [129]. Epple [125] revised the potential health risks of nanoscopic calcium phosphates, including HAp. The author reported that when HAp particles are ingested, they pass through different digestive organs, namely the stomach and gut. In the stomach, the complete dissolution of the HAp is achieved because it enters into contact with the very low stomach pH (around 1–2). In these high-acid conditions, the HAp particles are dissolved, losing their original chemical structure and shape [70,114,125]. Ramis et al. [130] report that HAp nanoparticulates are rapidly or even instantly dissolved into Ca^2+^ and PO_4_^2−^ ions when added to a simulated gastric fluid (pH around 1.2). The recently disrupted HAp then enters the gut, where the pH becomes basic. The small intestine absorbs calcium ions, but HAp recrystallisation may occur locally [125]. Under these new physiological conditions and in the presence of a high phosphate and calcium ion concentration, HAp may precipitate if its solubility is exceeded and the high concentration of biomolecules does not inhibit the nucleation; i.e., different forms (amorphous calcium phosphate) and sizes can be generated [125].

After concerns were raised about the use of HAp in food, mainly due to pristine particle size and shape, other studies have been conducted to evaluate its safety. For example, Schoepf et al. [128] studied the presence of nano-HAp in six different infant formula samples. Although crystalline needle-shaped HAp was detected in half of the samples, these authors documented a rapid dissolution of the HAp at low pH conditions, similar to simulated biological acidic gastric fluids. Ramis et al. [130] achieved similar results with rod-like HAp particles in acidic fluids. These authors also reported that rod-like HAp has no cytotoxicity effects when in contact with human gingival epithelium tissue. This work is of significant importance to understanding the behaviour of HAp with different shapes in the nano-scale range in the human organism. HAp safety was reinforced in this work as it reports the instantaneous dissolution of calcium phosphates into their ions under acidic conditions. This conclusion agrees with the Epple [125] statement that rules out any adverse health effects derived from exposure to HAp and other calcium phosphates in food products.

In emulsion stabilisation, HAp tends to form O/W systems. Some studies reported the capacity of HAp to be used in PEs or PE-derived products. However, their use in stabilising food-grade emulsions is still scarce. Table 2 summarises the results of work using HAp to produce PEs.

From the information in Table 2, as reported in Table 1, it can be perceived that some emulsions are composed of non-food-grade components, especially oil phases (DCM). Considering this information, it is important to guide studies towards the use of more realistic emulsion systems, especially in terms of oil phases and surface modifier agents.

In most cases, different surface modifiers or polymers dissolved in the oil phase are used to achieve the desired final goal, i.e., using PEs as templates for microencapsulation. The most-used polymers are poly(ε-caprolactone) (PCL) and poly(L-lactic acid (PLLA). Fujii et al. [127] and Fujii et al. [131] produced microspheres prepared via the PE solvent (dichloromethane) evaporation method with HAp and PCL. In this work, HAp was considered an adequate Pickering stabiliser in the production of PCL-coated HAp particles only when the interactions between carbonyl/carboxylic acid groups of the polymer and solid particles were favoured. This factor was crucial in obtaining stable and well-defined droplets and subsequent microspheres. The effect of HAp and PCL concentration and homogenisation rate on droplet size distribution was studied, concluding that an increase in PCL concentration from 1 to 10 wt% increased the average diameter of the microspheres from 15 to 38 μm. An increase in the homogenisation stirring rate from 14,500 to 30,000 rpm led to smaller emulsion droplets.

Another polymer often combined with HAp is PLLA (poly(L-lactic acid). HAp-stabilised PEs were produced with PLLA dissolved in dichloromethane, the oil phase. Emulsion type and stability were studied by Zhang et al. [26]. They showed that the interaction between HAp and PLLA has an essential role in PE stabilisation since it promotes the adsorption of the HAp particles at the oil–water interface. Microspheres produced through O/W and porous materials through W/O PEs were obtained after PE curing via an in situ solvent evaporation method [26].

Zhang et al. [137] studied the effect of HAp modified by stearic acid dispersed in different solvents (water, ethanol or dichloromethane) using an oil phase containing PLLA. PE stability and the ability to originate different structures after solvent evaporation was inspected. The authors noted that HAp surface modification in different solvents played an important role in the PE stabilisation and microstructure of the cured materials. HAp modified with stearic acid in ethanol increased the emulsion stability and allowed the obtaining of cured materials with uniform pore size. Stable O/W and W/O PEs were prepared using unmodified HAp and stearic-acid-modified nanoparticles (10 wt%, HAp-basis), respectively. The PE inversion was related to the enhanced hydrophobicity of HAp particles after surface modification [137]. Song et al. [139] studied the factors influencing the stability of PEs stabilised by HAp and non-ionic surfactants. In this work, the hydrophobicity of HAp particles was enhanced by surface modification using stearic acid dispersed in ethanol; PLLA and span 80 were dissolved in the oil phase. For moderated span 80 concentrations, emulsion stability was improved, and the porosity of the cured materials increased. However, when span 80 concentration increases, the emulsion undergoes a phase inversion from O/W to W/O.

The formation of PEs stabilised via HAp particles and polystyrene (PS), dispersed in the water and oil phases, respectively, was studied by Okada et al. [27]. The authors investigated the influence of polymer end groups (PS-CH_3_, PS_H_-COOH, PS_M_-COOH and PS_L_-COOH; subscribed letters mean high, medium and lower molecular weight, respectively) on the formation of HAp-stabilised droplets/microspheres, reporting that the end groups have an important role in emulsion stabilisation and in controlling the morphology of the microspheres prepared by solvent evaporation. They observed that lower molecular weight PS-COOH enhanced the interaction between HAp nanoparticles at the oil/water interface, improving emulsion stability. Additionally, the authors observed that the product’s structure after evaporation changes from a spherical to a deflected shape with decreasing PS-COOH molecular weight. Tham and Chow [138] modified the HAp surface properties through the incorporation of salt and amphiphilic agents such as cetyltrimethylammonium bromide (CTAB) and propyl gallate (PG). The pH was also varied to study the effect on HAp interfacial adsorption. In this way, the authors could control the droplet morphologies, which ranged from spheres and dumbbells to plated-shape and deflated-sphere, by changing the interfacial adsorption behaviour of HAp solid particles.

In the work of Hu et al. [140] and Rodríguez et al. [141], PEs were produced with HAp particles modified by sodium oleate and stearic acid, respectively, with no polymers or surfactants added to the oil phase. Hu et al. [140] observed a phase inversion from O/W to W/O and then back to O/W, induced by increasing surfactant concentration. Rodríguez et al. [141] successfully used W/O PEs stabilised by modified stearic acid HAp particles (HAP-ST) as a venom treatment, substituting for the traditional Freund’s emulsified vaccines, which have limited use due to tissue damage issues. The produced HAp-ST PEs have similar rheological behaviour using lower oil content and surfactant concentration, resulting in similar adjuvant activity and lower adverse side effects.

An antibacterial product was produced using HAp-stabilised PEs as the template for poly(melamine-formaldehyde) (PMF) capsule production [142]. Firstly, the authors prepared an O/W emulsion stabilised with HAp, and thereafter the hybrid spherical and rough microcapsules were formed by in situ polymerisation of PMF at the HAp surface. They reported the production of capsules with good thermal stability until 245 °C and with long-term antimicrobial activity. For example, after storage for 60 days, the bacterial inhibition rate of the microcapsules against *S. aureus* and *E. coli* was 86.5% and 83.3%, compared to the 99.6% and 98.4% observed at the initial time, respectively [142]. 

In addition to the production of microspheres through the PE route, the production of scaffolds has also been attempted. In this case, polymers dissolved in the oil phase to improve the interaction of the HAp particles were used. The scaffolds were prepared using solvent evaporation from PEs templates. Composite PCL–HAp scaffolds produced via PEs were developed for implementation on osteoblast cell proliferation by Samanta et al. [132], while Hu et al. [133] studied the production of scaffolds from the combination of HAp modified by PCL. In this last case, scaffolds with adjustable grid-like structures were fabricated by solvent evaporation of 3D printed W/O high internal phase emulsions (HIPEs), revealing a potential for anti-inflammatory drug release and efficient support for cell adhesion and proliferation. Hu et al. [135] fabricated scaffolds with HAp and PLLA for anti-inflammatory (ibuprofen) drug release. They showed that scaffolds exhibited a sustained release of ibuprofen, which increased for the higher HAp concentrations and pH, with the optimal conditions being 4 *w*/*v*% of HAp and pH of 7.4. The release profiles were well-fitted to the Higuchi model. Liu et al. [134] investigated the combination of HAp with PLLA and PCL for scaffold fabrication. HAp modified with alginate and coated with PLLA was also used for scaffold development by Hu et al. [145].

In recent studies, stable O/W PEs using HAp solid particles as Pickering stabilisers, without combination with polymers or emulsifiers, were produced by Ribeiro et al. [25] and Ribeiro et al. [144]. The authors reported that, for HAp contents above 5 wt%, enough solid particles were available to stabilise the oil surface and develop a particle network in the continuous phase, enabling emulsion stabilisation for 2 months. The authors produced PEs using conventional equipment, namely a rotor-stator, overcoming the limitations of this device (lack of temperature and droplet size control) by producing PEs in a continuous mode using a static mixer device, NETmix (described in Section 4.6).

The main advantage of PEs is their stability against coalescence and Ostwald ripening, making them interesting systems to use for encapsulating and delivering bioactive compounds [146]. Recently, HAp particles were used to produce vitamin E-loaded PEs. They were subjected to in vitro digestion and bioaccessibility studies, then used to produce fortified products [114]. The authors reported that vitamin E-loaded PEs stabilised by n-HAp particles disrupted under gastric conditions, forming aggregates under the intestinal environment. Additionally, when these PEs were incorporated into food matrices, namely gelatine and milk, vitamin E bioaccessibility increased significantly (3.3 and 6 times higher, relative to the corresponding bioaccessibility of non-incorporated PEs), indicating the positive effect on the food matrix in terms of bioaccessibility [114].

## 4. Preparation of Pickering Emulsions—Production Processes

The process by which two immiscible liquids are converted into an emulsion is known as emulsification, while homogenisation is the act that makes emulsion droplets uniformly distributed. Emulsification and the subsequent homogenisation are usually carried out using mechanical devices known as homogenisers [1]. The production of emulsions requires external mechanical energy to break up the dispersed phase into small droplets, homogenising this phase throughout the continuous phase [147]. The most common types of equipment to prepare PEs are high-shear mixers and high-pressure and ultrasonic homogenisers (see Table 1 and Table 2). These types of devices allow the preparation of small amounts of PEs, and, subsequently, the production is often performed in batch mode. However, techniques such as membrane homogenisers and microfluidisers have been applied to prepare PEs. A brief description of each technique is given next.

### 4.1. High-Shear Mixers

**Figure 4:**
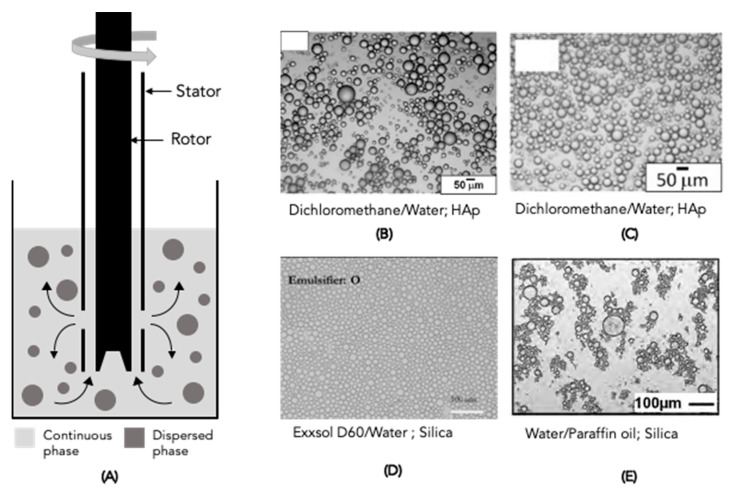
Schematisation of rotor-stator device (**A**), and some examples of PEs ((**B**) [127], (**C**) [131], (**D**) [44] and (**E**) [100]). (Reprinted from [127], Copyright (2012), with permission from Elsevier; Reprinted from [131], Copyright (2013), with permission from Taylor and Francis; Reprinted from [44], Copyright (2017), with permission from Elsevier; Reprinted from [100], Copyright (2010), with permission from Elsevier).

High-shear mixers, which are a type of rotor-stator device, are the most used devices for homogenising oil and aqueous phases [1]. In PE production, this device is the most used [2]. It consists of a rotor and stator with blades and openings, respectively; usually, the emulsification takes place in a suitable container (Figure 4A). When the rotor rotates, a depression is created, drawing the liquid in and out, resulting in liquid circulation [2]. The shear force between the rotor and stator and the high liquid acceleration causes the droplet size reduction of the dispersed phase [2]. For PE production, the oil and water phases can be added at the beginning or added sequentially to improve the emulsification in terms of the droplet size [1,2].

According to the literature, high-shear mixers are the most applied devices to obtain PEs. However, different operating conditions, such as rotation speed and time, are used by different authors to produce PEs with different oil and aqueous phases. Generally, the rotation speed ranges from 2500 to 30,000 rpm while the rotation time ranges from 30 s to a few minutes, as can be seen in Table 1 and Table 2. The lack of control during emulsification is the main disadvantage of this device, leading to the obtaining of PEs with broad droplet size distributions ranging from a few to hundreds of microns [2]. For example, Björkegren et al. [44] investigated the energy input during the emulsification process and concluded that it is an essential parameter in decreasing PE droplet size. The increased stirring speed from 10,000 to 20,000 rpm led to a decrease in the droplet mean diameter from 10 µm to 4 µm. Cui et al. [47], Santini et al. [43], Binks and Yin [104] and Song et al. [139] provide other examples of work producing PEs with a rotor-stator device using different solid particles. Recently, Ribeiro et al. [25] produced PEs stabilised by n-HAp solid particles using a rotor-stator device. PEs were produced at 11,000 rpm for 6 min, using a thermostatic bath to control temperature during emulsification, overcoming temperature-rising constraints. Figure 4B–E show images of PEs produced using a high-shear mixer, where the droplet size range can be observed. However, a problem with an increase in stirring speed is the rise in temperature; thus, other techniques have been proposed to produce PEs with better control.

### 4.2. Ultrasonic Homogeniser

**Figure 5:**
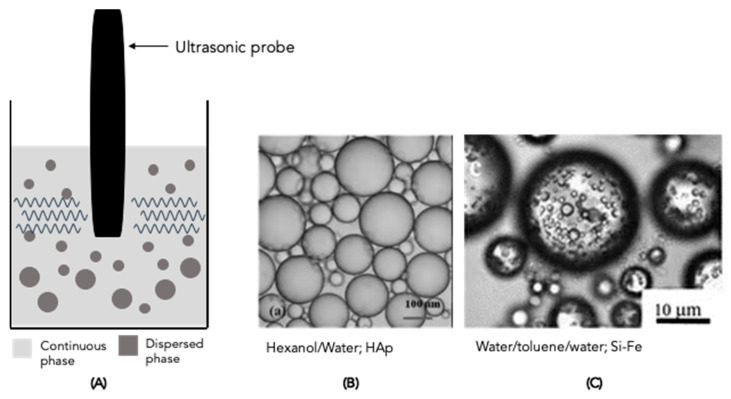
Schematisation of the ultrasonic device (**A**), and some examples of PEs ((**B**) [143] and (**C**) [105]). (Reprinted from [143], Copyright (2019), with permission from Elsevier; Reprinted from [105], Copyright (2013), with permission from Taylor and Francis).

Ultrasonic technology is another method used to produce PEs, where the ultrasonic probe is the most commonly used configuration [2]. It consists of a titanium probe that vibrates due to a transducer containing a piezoelectric crystal, which converts electric energy to very high-frequency mechanical motion (Figure 5A). The probe transmits ultrasonic energy to the surrounding sample, inducing emulsification mainly through cavitation and turbulent effects [1,2]. Ultrasonic devices use high power to interact with the materials; however, in PEs, which use sensitive particles, the use of ultrasonic homogenisers requires some caution, since they can expose the emulsion to high-intensity ultrasound power [1] as well as to local high temperatures [148], promoting physical and/or chemical degradation of the particles [1]. For PE production, the emulsions are subjected to amplitudes that range from tens to hundreds of watts and a few minutes are usually applied to prepare the emulsion. In general, these are the main parameters affecting emulsion droplet size, and the use of optimised parameters can lead to the minimum droplet size. However, it is important to point out that the optimal parameters strongly depend on the system composition, requiring a well-planned experimental design.

Previous work has reported PEs produced by ultrasonic emulsification with various solid particles, including silica and HAp [56,105,135,138,143]. Some optical images of the obtained PEs are shown in Figure 5B,C. However, according to the data presented in Table 1 and Table 2, few studies have dealt with the production of PEs by this emulsification method when compared with others, such as the rotor-stator. For this reason, most of the work reported in the literature does not make an intensive study of the influence of ultrasonic emulsification parameters on emulsion properties, namely stability.

### 4.3. High-Pressure Homogeniser

**Figure 6:**
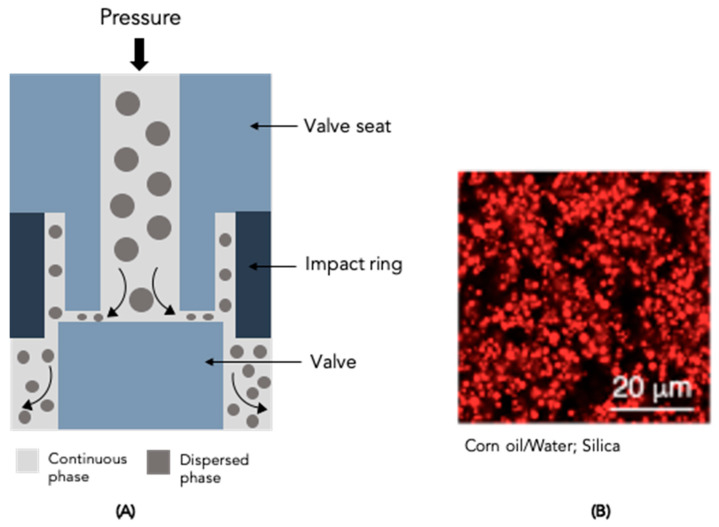
Schematisation of high-pressure device (**A**), and an example of PE ((**B**). (Reprinted from [106], Copyright (2016), with permission from the American Chemical Society).

High-pressure homogenisers are the most common devices used in the food industry to prepare CEs [1]; however, according to Table 1 and Table 2, this homogeniser is seldom used in PE preparation. Before using this homogenisation technique, it is recommended to obtain a coarse emulsion [1]. A schematic diagram of a high-pressure homogeniser is shown in Figure 6A.

The pressure increases due to a high-pressure pump and the coarse emulsion is injected into a homogenising nozzle of small size, which disrupts the droplets, producing a fine emulsion [2]. The fluid characteristics (e.g., viscosity) and nozzle design are the main parameters responsible for the disrupting of the droplets [1]. Different types of nozzles have been designed and fabricated to increase the efficiency of droplet disruption. Moreover, the emulsion droplet size can also be reduced through repeated cycles of the PE in the homogeniser [1,2]. According to the data shown in Table 1 and Table 2, PEs with lower droplet sizes can be obtained by varying the pressure from tens to hundreds of MPa and/or by repeatedly recirculating the emulsion through the device. Köhler et al. [60] studied the impact of pressure on emulsion formation with silica particles. They reported a reduction of ~40 µm to ~10 µm in droplet size with an increase in pressure from 350 to 800 bar, after which no further droplet break-up was observed. Eskandar et al. [90] and Alison et al. [106] (Figure 6B) also reported the use of a high-pressure device to produce PEs; in each work, distinct operation parameters were used, suggesting that the pressure and number of cycles have a crucial role in emulsion droplet size. These parameters can be adjusted according to particles and oil type used.

### 4.4. Microfluidizers

**Figure 7:**
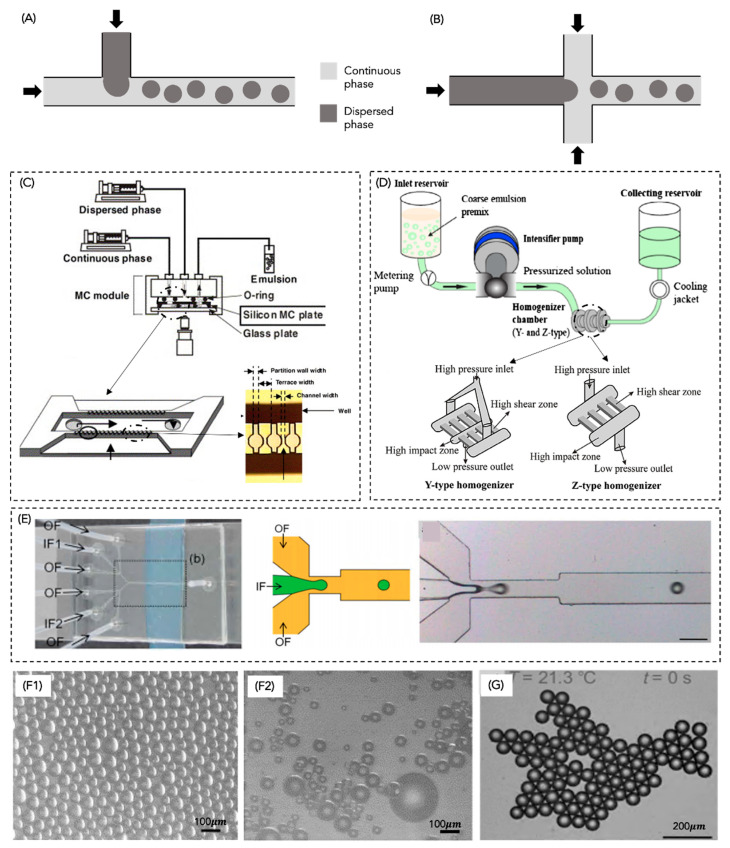
Schematisation of microfluidizers: (**A**) T-junction and (**B**) flow-focusing. Microfluidic technologies used in PE production ((**C**) [89], (**D**) [149] and (**E**) [150]). Some optical images of PEs obtained from microfluidizers: (**F1**) PE obtained from microfluidic device (**C**), and (**G**) PE obtained from microfluidic device E. (**F1**,**F2**) compare PEs produced by microfluidic device and rotor-stator, respectively. (Reprinted from [89], Copyright (2005), with permission from Elsevier; Reprinted from [149], Copyright (2016), with permission from Elsevier; Reprinted from [150], Copyright (2016), with permission from the Royal Society of Chemistry).

Microfluidic devices consist of microchannels with specific and well-defined geometry in which fluids circulate [2]. Fluids are introduced into the device, which is accelerated to a high velocity inside the channels through a pump, promoting an impinging upon each other. When the two fluid streams collide, high disruptive forces are generated, causing the mixture of the dispersed and continuous phases, as well as the break-up of the droplets, upon which the PE is produced [1]. There are two main types of microfluidizers, single-inlet (e.g., T-junction) and double-inlet microfluidizers (e.g., flow-focusing). In T-junction devices (Figure 7A), the dispersed phase is forced to flow through a small orifice into the perpendicularly flowing continuous phase.

In contrast, in the flow-focusing devices (Figure 7B), the dispersed phase is focused on two perpendicular streams of the continuous phase from both sides [2]. Through this method, PE droplet size can be controlled by changing the flow rate or by changing the channel geometry. However, changing the channel geometry is often unfeasible and/or expensive. This method has been commonly applied to CEs, but only recently applied to the production of PEs, and very few scientific studies were found.

Xu et al. [89] produced O/W PEs with silica through a microfluidizer (Figure 7C). The emulsions produced through a microchannel method (Figure 7(F1)) allowed the obtaining of well-defined droplets and emulsions with high stability (over several months) when compared with ones produced by the traditional rotor-stator method (Figure 7(F2)). Sun et al. [150] produced O/W PEs using the microfluidic device shown in Figure 7C, achieving stable droplets of very similar diameter, as shown in Figure 7G. Other microfluidic devices have been reported in the literature that enable the production of PEs without high-shear forces, such as the case shown in Figure 7D [149,150,151]. This technique for producing emulsions has been gaining interest in the scientific community since it offers a simple preparation and precise control of emulsion droplets.

### 4.5. Membrane Homogeniser

**Figure 8:**
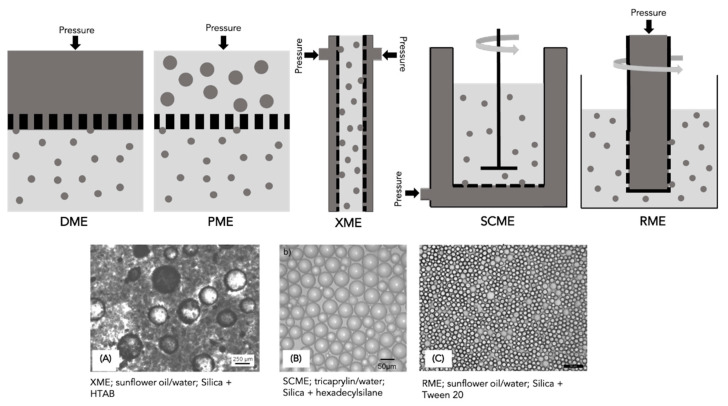
Schematisation of membrane emulsification. DME: direct membrane emulsification; PME: premix membrane emulsification; XME: crossflow membrane emulsification; SCME: stirred-cell membrane emulsification; and RME: rotational membrane emulsification. Some examples of PEs obtained from XME—(**A**) [103], SCME—(**B**) [97] and RME—(**C**) [102]. (Reprinted from [103], Copyright (2016), with permission from Elsevier; Reprinted from [97], Copyright (2017), with permission from the American Chemical Society; Reprinted from [102], Copyright (2019), with permission from Elsevier).

In the membrane emulsification technique, the dispersed phase is pressed through a porous membrane that contains well-defined pores into a continuous phase that usually contains the Pickering stabiliser [97]. According to the composition of the dispersed phase, the PE production through porous membranes can be divided into direct membrane emulsification (DME) and premix membrane emulsification (PME) (Figure 8) [2]. In DME, the emulsion is formed from initially separated oil and water phases where the dispersed phase is pressed through the porous membrane into the continuous phase; in PME, the coarse emulsion is pressed through the membrane and involves mainly a reduction in droplet size [1,2]. DME techniques have been upgraded, and three main types can be considered: crossflow membrane emulsification (XME), stirred-cell membrane emulsification (SCME) and rotational membrane emulsification (RME) (Figure 8) [2]. Both XME and SCME are similar to DME, but while in XME the fluid flows along a channel formed by the membrane, in SCME, the membrane is placed inside a stirred vessel [2]. For RME, the main characteristic is that the membrane is not stationary. For SCME and RME, agitation improves droplet detachment from the membrane, inducing a smaller droplet size formation [2]. The membranes can be designed and manufactured with different pore sizes that will induce different droplet sizes in the emulsion, and with a different polarity that should be carefully selected since it determines the type of emulsion [1]. However, this technique has only been used in research; its use is not common in the food industry due to low-volume throughputs [1].

Yuan et al. [59] used two types of silica particles as O/W emulsion stabilisers and compared XME and RME PE production, obtaining highly stable emulsion with narrow droplet size distributions. Yuan and Williams [103] produced co-stabilised PEs, which can guide the formulation of complex multi-functional particulates using XME and RME techniques. In both cases, depending on the oil flux rate and membrane speed, droplet mean diameter ranged from 10 to 200 µm. Furthermore, the authors reported that the co-stabilised emulsion has a lower mean diameter than the surfactant-only stabilised emulsion. The authors observed that higher stirring speed aided droplet breakup. Manga and York [97] also used SCME to prepare O/W PEs solely stabilised by silica particles. The authors achieved optimal conditions, in terms of oil flux rates (6 L/m^2^h), paddle stirrer speeds (1250 rpm) and oil volume fraction, to control droplet size and size distribution. Droplet size increased with the oil volume fraction increase and ranged from 40 to 170 µm. RME was used to produce silica-stabilised tricaprylin/water PEs with controlled droplet size, narrow polydispersity and highly stable emulsion [96]. The effect of the membrane rotational speed was tested, and the reported data showed a crucial influence on decreasing droplet size since it induces an easier detachment of the droplets from the membrane. Sun et al. [152] and Arkoumanis et al. [102] produced stable O/W PEs with small droplet sizes and narrow distribution via RME. In this context, Sun et al. [152] controlled the mean diameter of PE droplets through membrane pore size. They showed that by maintaining other emulsification parameters and increasing the pore size from 2.5 to 9.2 μm, the emulsion droplet size increased from 10 ± 0.5 μm to 50 ± 5.3 μm, concluding that the emulsion droplet size is normally 3−9 times larger than the membrane pore size [152]. Arkoumanis et al. [102] produced PEs around 12 μm with a membrane pore size of 6 μm. Figure 8A–C compare PE optical images produced by XME, SCME and RME techniques.

### 4.6. Static Mixers

High-shear mixers and high-pressure and ultrasonic homogenisers use high-shear mechanical forces, requiring high-energy inputs to produce emulsions. The main limitation is the lack of control, mainly in the droplet size, during the emulsification process [97]. However, they are the most used devices for achieving an adequate emulsification step. Recently, and as an alternative to these devices, other techniques have been gradually developed, such as membrane and microfluidic devices, and subsequent applications to produce PEs have been studied [23,97,102]. Increasing of production scales has led to a search for other types of devices.

The use of static mixers has been increasing in industrial applications mainly due to their unique advantages, such as mixing, heat transfer capabilities and operation in semi- or continuous mode [153]. These devices appear as an alternative to the traditional mechanical mixers for mixing immiscible liquids, homogenisation of solid particles and/or for heat and mass transfer improvement. Static mixers divide and redistribute streamlines in a sequential form and mixing between the fluids is ensured by the flow energy; thus, static mixers do not require external power, but just the power for pumping the fluids through the mixer [153].

Muruganandam et al. [154] produced O/W emulsions using an SMX static mixer (nine perpendicular Teflon elements assembled against each other) and studied the impact of the dispersed phase concentration, flow rate and operating time on emulsion droplet size. The authors reported a decrease of the Sauter average diameter of oil droplets from 8 to 4 µm with an increasing Reynolds number (Re), and a constant diameter around 4 µm when increasing the concentration of the dispersed phase from 1:100 to 1:25.

**Figure 9:**
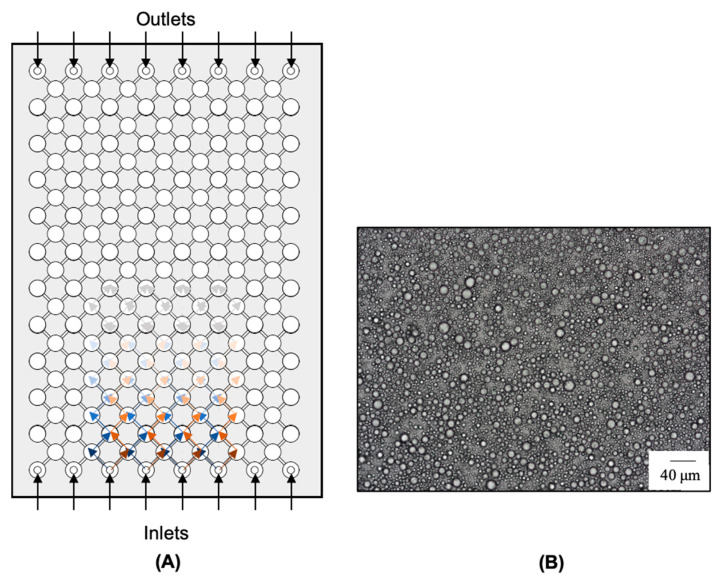
Schematisation of the mesostructured static mixer—NETmix (**A**) with the representation of the fluid streams inside of the reactor, and an example of PE produced via NETmix ((**B**). (Reprinted from [144], Copyright (2021), with permission from Elsevier).

More recently, NETmix, a mesostructured static mixer (Figure 9A), was applied to produce PEs. Ribeiro et al. [144] developed PEs in continuous mode considering an industrial perspective with n-HAp-stabilised PEs, and their size can be controlled depending on the Re and number of cycles used. The authors showed a reduction in the average droplet size, increasing the Re or the number of cycles; the minimum average droplet size obtained was around 7 µm when using 17 cycles and Re = 400 (Figure 9B). For PE production, NETmix ensures easy control of the parameters affecting mixing, providing good reproducibility among assays, not limiting the production volume, reducing the production time and allowing continuous mode production [144].

The higher mixing capacity of static mixers compared to currently commercially available devices enables a high potential for mixing two immiscible liquids [144,153,154], achieving the desired objective, mainly in terms of droplet size and lower production costs. Static mixers such as SMX and NETmix can offer a high mixing efficiency when compared to traditional mechanically stirred vessels. Static mixers enable well-localized mixing points, which promote an easily reproducible emulsification step. This should be taken into consideration because, in most cases, it can be a decisive criterion for the overall process performance. However, in the PE field, the number of publications in the literature concerning these types of devices is still limited when compared to the others.

Table 3 lists and describes the main production processes used for PEs. Overall, the choice of the device used for emulsification depends on various factors, including the scale and the volume of the emulsion, the physicochemical properties of the phases, the desired droplet size distribution and costs, as well as their specific advantages and disadvantages [1].

## 5. Pickering Emulsions for Food Applications

Food has an essential role in human nutrition and health. It represents the primary source of energy and essential nutrients, such as vitamins, minerals and bioactive phytochemicals [155]. Currently, there is a growing demand for food safety and healthy products, leading to an investment in research and development of new processes and products to satisfy consumers’ concerns.

PEs’ popularity in food applications has increased in recent years. This is mainly due to their high stability compared to conventional systems, as well as to the wide range of stabilising particles [29]. These advantages make PEs good candidates for delivery systems since stimuli-responsive PEs can function as a route to triggered release, ensuring the protection and/or delivery strategies of the various bioactive compounds [115].

Most of the research on PEs has been conducted with safe inorganic particles such as silica, which is used as a model Pickering stabiliser in the design and development of new PEs [24]. This knowledge allows the development of potential applications for different fields, including the food industry. However, some challenges related to the implementation of PEs in the food sector must be taken into account to ensure the preservation and robustness of these systems. For example, electrolyte concentration, pH and compatibility with other food ingredients are some examples which could affect PE structural integrity [70,156].

### 5.1. Emulsifier Substitution in Food

PEs have arisen in an attempt to develop new alternatives to conventional emulsions and to respond to consumers’ issues and/or concerns since the emulsifiers used have been related to some harmful health problems. Particles such as flavonoids, polyphenols and proteins, to which some beneficial health effects after consumption can be attributed, can be used as Pickering stabilisers, improving emulsion functionality [157,158].

Flavonoids are naturally present in fruits and vegetables, and their consumption has been associated with antioxidant and anti-inflammatory functionalities in the body [159]. Recently, Luo et al. [158] investigated three types of flavonoids (tiliroside, rutin and naringin) as stabilisers of O/W Pickering emulsions. Stabilisation was dependent on pH, increasing for higher values. Rutin was found to improve the oxidative stability of a whey protein-stabilised O/W emulsion during 1 month of storage at 50 °C, as the presence of rutin was also related to the stability improvement, namely avoiding coalescence [160]. The authors also reported that a significant proportion of rutin was adsorbed at the oil–water interface, either partially replacing the protein or by co-adsorbing with it, forming a densely adsorbed layer at the interface that can be antioxidant, and hence protect the emulsion against chemical degradation.

Polyphenols are abundant micronutrients in fruits and vegetables, and there is evidence of their important role in the prevention of degenerative diseases such as cancer and cardiovascular diseases [161]. Recent studies have shown that they can act as Pickering stabilisers at the water–oil interface [157]. Zembyla et al. [157] propose a novel way to stabilise water droplets via interfacial complex formation through water-insoluble polyphenol crystals and protein. The authors observed that complex polyphenol crystals (curcumin or quercetin) and whey protein adsorb at the interface and provide stabilisation of water droplets for 21 days. No significant differences in stabilisation time were detected for curcumin and quercetin. The mean droplet diameter remained stable over storage, with 22 µm and 27 µm for curcumin and quercetin, respectively [157].

Proteins in emulsion systems also entail potential health benefits which may arise from the consumption of bioactive proteins or the formation of bioactive peptides post-ingestion [162]. Lactoferrin has aroused interest for its various implications for biological functions, such as antioxidant and antimicrobial activities [163]. Shimoni et al. [162] studied the ability of lactoferrin to stabilise O/W emulsions. They found that using protein nanoparticles increased the stability of coarse emulsions but not the fine emulsions produced by high-pressure homogenisation. The combination of lactoferrin with alginate and *i*-carrageenan improved emulsion stability against proteolysis during in vitro gastric digestion compared to native lactoferrin.

Recently, PEs have been used as substitutes for conventional emulsifiers in food products such as mayonnaise. In this case, the solid particles were used as an alternative to egg yolk and contributed to developing disruptive products with vegan characteristics. In this context, Lu et al. [164], Akcicek et al. [165], Ghirro et al. [166] and Li et al. [167] studied the possibility of using PEs stabilised by apple pomace particles, gum nanoparticles, curcumin-based solid dispersion particles and pea protein isolate microgels, respectively, for the development of edible mayonnaises. The stability of the emulsions during storage and against different environmental stresses and rheological properties was studied to validate the use of solid particles.

### 5.2. Fat Reduction or Substitution

Obesity and cardiovascular disease are worldwide health problems, which, in most cases, are related to excessive intake of saturated fatty acids commonly encountered in processed foods [168]. In this way, there is an increased interest in substituting saturated fatty acids with healthier alternatives. A possible approach is the use of PEs as templates to develop low-viscosity liquid oil into soft gels [169] or high internal phase Pickering emulsions (HIPEs) [14,170]. Gao et al. [169] developed a zein protein–sodium stearate complex-based O/W PE, a suitable process for producing oil gels. PEs with sodium stearate and zein (10 mM and 0.5 wt%, respectively) were revealed as a homogeneous and translucent gel without oil leakage. W/O HIPEs can provide interesting textures and can be used to reduce trans and/or saturated fat content in food products. HIPEs are characterised by their high dispersed phase volume ratio (0.74 or higher), showing droplets tightly packed with the continuous phase acting as a liquid film, giving these emulsions a highly viscous characteristic [170]. In HIPEs, the particles can form a particle–particle network in the space between the droplets, playing the role of a “structuring agent”; particles can effectively adsorb and become irreversibly anchored at the oil–water interface to prevent droplet aggregation by creating steric hindrance [69,171]. HIPEs can serve as a direct substitute for oil to decrease fat intake, satisfying the consumer’s demand for healthier products.

Another alternative is the replacement of saturated fats with polyunsaturated fats, which are known to be healthier. In this line of thought, PEs are advantageous due to their high physical and chemical stability since polyunsaturated fats are highly susceptible to lipid oxidation [16,95]. Kargar et al. [16] and Kargar et al. [95] are some examples of work focusing on the oxidative stability of PEs stabilised with silica, microcrystalline cellulose and modified starch. It was shown that microcrystalline cellulose particles were able to reduce lipid oxidation more effectively than modified starch particles, which was attributed to the ability of microcrystalline cellulose to scavenge free radicals due to their negative charge, and to form thicker interfacial layers around oil droplets [16]. Kargar et al. [95] reported that when sodium caseinate is dispersed in the continuous phase, a reduced lipid oxidation at pH 7 was found due to its metal chelating ability. In addition, the results showed that emulsions stabilised with silica particles (at pH 2) inhibit lipid oxidation to a greater extent than emulsions stabilised with Tween 20. Particles demonstrated the ability to separate pro-oxidants present in the continuous phase from hydroperoxides located at the droplet interface [29].

The high particle concentrations in the continuous phase may have a filler effect or function as a fat substitute [29]. As an example, Skelhon et al. [84] created healthier chocolate, which was infused with fruit juice using a W/O PE. The emulsion was produced with silica–chitosan particles. The authors replaced ~50 wt% of the chocolate fat with fruit juice in the form of emulsion droplets.

### 5.3. Encapsulation of Active Compounds and Development of Functional Foods

Functional foods refer to those foods that have an active or functional compound which is not naturally present. It should add benefits or functions that the food would otherwise not have [155]. Vitamins (A, E, D and K), fatty acids (ω-3), dietary fibre, proteins and natural bioactive compounds such as polyphenols are some examples of the active compounds that are usually used to fortify food products [29]. These active compounds, which have mostly antioxidant capabilities, are incorporated in the food matrix to provide physiological benefits, preventing some diseases such as heart disease, hypertension or inflammatory processes [172,173].

Lipophilic bioactive compounds, specifically lipophilic vitamins, have reduced solubility in water and can also be unstable in adverse conditions [115,174]. Thus, O/W emulsions are among the most relevant and versatile encapsulating and delivery systems for these compounds in food applications. PEs, which are known to have excellent physicochemical stability, can serve as encapsulating systems for hydrophobic compounds, also improving their stability and bioaccessibility [29]. The encapsulation technique allows the production of a barrier that protects sensitive compounds from the hostile environment; in this way, it can lead to effective absorption of the active compound in the body [175].

In recent years, various works have reported the role of PEs in emulsion stability, encapsulation and release of bioactive compounds. For example, Tikekar et al. [99] produced O/W PEs with silica particles and used the emulsions as controlled-released vehicles for curcumin. Curcumin, which is a hydrophobic polyphenol, has significant antioxidant and anti-inflammatory properties, becoming rapidly unstable under unfavourable environmental conditions [176], needing to be encapsulated to maintain its bioactivity. Tikekar et al. [99] reported that silica-stabilised PEs have ~80% of curcumin retention after simulated gastric digestion and ~60% of curcumin release after two hours of simulated intestinal digestion.

Zhou et al. [177] studied the use of oregano essential oil in PEs stabilised by cellulose nanocrystals for antimicrobial essential oil delivery. Results showed that the oregano essential oil PEs inhibited the growth of four microorganisms (*Escherichia coli*, *Staphylococcus aureus*, *Bacillus subtilis* and *Saccharomyces cerevisiae*) by destroying the integrity of the respective cell membranes.

Vitamin D_3_ is a fat-soluble vitamin essential for humans, but its synthesis is only achieved after sun exposure [115]. PEs emerge as a good approach to increase the use of this vitamin. Winuprasith et al. [115] studied the encapsulation of vitamin D_3_ using PEs through mangosteen cellulose particles. The authors reported that the vitamin could be digested and absorbed in the gastrointestinal tract with relatively low levels of solid particles. However, when large amounts of solid particles (0.7%) were used, the particles function as a “protective shell”, inhibiting the release of the vitamin in the gastrointestinal tract.

Another example of PEs used in the food area is reported in the work of Stratulat et al. [178], which developed an approach with calcium caseinate and lecithin particles for vitamin D_3_-fortified cheese. The obtained results showed a recovery level of vitamin D_3_ of around 84%, maintaining its stability during 3 months of storage. Overall, the results indicate that the encapsulation of vitamin D_3_ in cheese, in the form of emulsified particles, increased its retention and stability in the curd and improved the chemical stability of fortified chess against oxidation.

Recently, Ribeiro et al. [114] used n-HAp Pickering emulsions as vitamin E carriers in gelatine and milk food applications. After incorporation, the PE droplets remained within their typical size and morphology. The authors reported better vitamin E bioaccessibility (3.3 and 6 times higher in gelatine and milk, respectively) after incorporating the emulsion in the food matrix, compared to the Pickering emulsion’s performance alone. This fact was attributed to the natural presence of macronutrients (fat and proteins) in food matrices, which can improve micellar phase formation.

## 6. Conclusions

Emulsions stabilised by solid particles, PEs, offer attractive advantages compared to CEs, which are stabilised by emulsifiers. The generally good inherent stability of PEs is their most important advantage, justifying the high interest they have gathered in different research and industrial fields over the past 20 years, leading to an increased number of applications. Although PEs have several advantages, some challenges related to formulation development for commercial applications, mainly in developing innovative food products, still remain. In this context, the study of parameters affecting emulsion stability is worthy of investigation.

Several solid particles have been investigated as Pickering stabilisers. However, a wide range of parameters must be taken into consideration to develop a stable PE: particle properties (e.g., size and shape), aqueous phase (e.g., pH, ionic strength) and oil phase (e.g., viscosity). In this context, safe inorganic solid particles can be used as Pickering stabilisers. Usually, they are well-stabilised particles with specific and constant sizes. Among them, silica particles are the most studied, but others can be used, such as hydroxyapatite. In this context, several studies should be explored to understand the mechanisms and parameters governing PE stability, namely the ones stabilised by non-spherical particles. Identifying how the nature, stability and application of solid particles are related to their structure/shape is necessary. It is also important to develop models to predict emulsion behaviour when non-spherical particles are used and understand the adsorption mechanisms at the interfacial surface. Additionally, to improve the knowledge of solid particles, it is important to study the toxicity and allergy of these materials in in vitro and in vivo environments. In terms of HAp, it is interesting to study particle behaviour in final product application and use in calcium supplementation.

PEs are predominantly prepared using high-shear techniques; although these technologies enable relatively fast production, they are characterised by a lack of control during production. Membranes and microfluidic devices appear to overcome the disadvantages of high-shear technologies producing PEs with controlled size of droplets and lower polydispersity. However, to answer the demand for PE industrialisation, devices such as static mixers are emerging in the PE field. These devices enable emulsion size control, high performance due to continuous production and process reproducibility. Thus, it is important to use this technology at the industrial level to increase production rates and make viable PE-derived food products.

Among the range of possibilities of PEs in the food industry is their use in the development of fat-reduced products and functional foods since they can serve as encapsulating carriers, for example, of hydrophobic compounds such as lipophilic vitamins. Considering this information, the application of HIPEs in the development of food products should be explored. In addition to PE functionality, rheological properties must be considered in the development of products with the desired appearance and organoleptic characteristics. In this context, the field of PEs needs to evolve and take a step forward to studying more realistic formulations and production processes for the industry, providing a route to develop healthier and safer food products.

## Figures and Tables

**Table 1:** Examples of PEs stabilised with inorganic food-grade particles.

Particle Characterisation				Emulsion Characterisation			Production		Ref.
Solid Particle	Surface Modification	Shape	Size	Water Phase	Oil Phase	Emulsion Type	Homogeniser	Rate Time Pressure Cycles	
Silica	n.a.	Spherical	30 nm	Water	Tricaprylin	O/W	Microfluidizer	n.d.	[89]
Silica	Lecithin or oleylamine	Spherical	7 nm	Water	Miglyol	O/W	High-pressure	500 1000 bar 5 cycles	[90]
Silica	Monoolein	Spherical	150 nm	Water	Vegetable oil	O/W	High-shear	8000 rpm 5 min	[91]
Silica	Sodium dodecyl sulphate	Spherical	12 nm	Water	n-dodecane	O/W	Rotor-stator	13,000 rpm 2 min	[92]
Silica	n.a.	Spherical	80 nm or 800 nm	Water	Ethyl acetate	O/W	XME; RME	n.a.	[59]
Silica	Tween 60; sodium caseinate; lecithin	Spherical	150 nm	Water	Vegetable oil	O/W	High-shear	8000 rpm 5 min	[83,93]
Silica	n.a.	Spherical	145 nm	Water	Hexadecane	O/W	Hand shaking	n.a.	[94]
Silica	n.a.	Spherical	100 nm	Water	Toluene	O/W	Ultrasonic	40% amplitude	[56]
Silica	n.a.	Spherical	12 nm or 200 nm	Water	Corn oil	O/W	High-pressure	350–1000 bar 1 cycle	[60]
Silica	n.a.	Spherical	12 nm	Water	Sunflower oil	O/W	Rotor-stator	7 min	[95]
Silica	n.a.	Spherical	800 nm	Water	Tricaprylin oil	O/W	RME	n.a.	[96]
Silica	n.a.	Spherical	10–12 nm	Water	Tricaprylin oil	O/W	SCME	n.a.	[97]
Silica	n.a.	Spherical	15 nm	Water	n-dodecane	O/W	Rotor-stator	13,000 rpm 3 min	[98]
Silica	n.a.	n.d.	8 nm	Water	Canola oil	O/W	High-pressure	600 bar 3 cycles	[99]
Silica	Sorbitan monooleate	Spherical	12 nm	Water	Paraffin oil	O/W	Rotor-stator	25,000 rpm 5 min	[100]
Silica	mPEG silanes; organosilanes	Spherical	13–70 nm	Water	Exxsol D60	O/W; W/O	Rotor-stator	10,000–20,000 rpm 4 min	[44]
Silica	Palmitic acid	Spherical	15 nm	Water	Hexane	O/W	Rotor-stator	10,000 rpm 10 min	[43]
Silica	Oleic acid	Spherical	5–10 nm	Water	Paraffin oil	O/W	Magnetic stirrer	2500 rpm 2 min	[42]
Silica	CTAB	Spherical	20 nm	Water	n-dodecane	O/W	Rotor-stator	7000 rpm 2 min	[101]
Silica; hydroxyl methyl cellulose	Tween 20; whey protein	n.d.	n.d.	Water	Sunflower oil	O/W	RME	n.a.	[102]
Silica + PS latex	SDS; HTAB; Tween 20	n.d.	n.d.	Water	Paraffin oil; ethyl acetate; sunflower oil	O/W	XME; RME	n.a.	[103]
Silica (1) or zirconia (2)	Dipropyl adipate	Spherical; n.d.	5–30 nm (1); 5–10 nm (2)	Water	n-dodecane	O/W	Rotor-stator	13,000 rpm 2 min	[104]
Clay (1); silica (2); Fe_2_O_3_ (3); oleic acid-coated Fe_2_O_3_ (4); microgel (5)	n.a.	Platelets (1); spherical (2,3,4); microgel (5)	1 × 30 nm (1); 5–30 nm (2); 5 nm (3,4); 220 nm (5)	Water	Styrene; toluene	W/O/W; O/W/O	Ultrasonic; Hand shaking	2 min	[105]
Silica/ chitosan	n.a.	n.d.	n.d.	Water	Sunflower oil; cocoa butter	W/O	Rotor-stator	11,000 rpm 2 min	[84]
Silica/ chitosan	n.a.	n.d.	n.d.	Water	Corn oil	O/W	High-pressure	1380 bar 7 cycles 2760 bar 1 cycle	[106]
Clay	SDS; DTAB; Pluronic	Spherical	9–50 nm	Water	Mineral oil	O/W	Rotor-stator	11,000 rpm 5 min	[107]
Calcium carbonate	n.a.	Cubic	~1 µm	Buffer solution	Sunflower oil	O/W	Rotor-stator	6000 rpm 2 min	[108]
Calcium carbonate	n.a.	Spherical; cubic; rod-like	~5 µm	Water	Soybean oil	O/W	Hand shaking	30 s	[33]
Calcium carbonate	Fatty acids	Spherical	80–100 nm	Water	Toluene	O/W; W/O	Rotor-stator	5000 rpm 2 min	[47]
Silicone resin	n.a.	Microbowl	2–2.5 µm	Water	n-dodecane	O/W	Vortex mixer	n.d. 2 min	[64]

CTAB—cetyltrimethylammonium bromide; DTAB—dodecyltrimethylammonium bromide; HTAB—hexadecyl trimethyl ammonium bromide; mPEG—poly(ethylene glycol) silane; n.a.—not applicable; n.d.—not defined; RME—rotational membrane emulsification; SCME—stirred-cell membrane emulsification; SDS—sodium-dodecylsulfate; XME—crossflow membrane emulsification.

**Table 2:** Main PEs produced with HAp as solid stabiliser.

Particle Characterisation			Emulsion Characterisation			Production		Use	Ref.
Surface Modification	Shape	Size	Water Phase	Oil Phase	Emulsion Type	Homogenizer	Speed/Time Pressure/Cycles		
PCL *	Rod-like	30 nm	Water	DCM	O/W	Rotor-stator	20,500 rpm 1 min	PE stabilisation	[127]
PCL *	Rod-like	30 nm	Water	DCM	O/W	Rotor-stator	14,500–30,000 rpm 1 min	PE stabilisation	[131]
PCL *	Fibril	23 × 140 nm	Water	DCM and DMF	W/O	Rotor-stator	15,000 rpm n.d.	Scaffolds fabrication	[132]
PCL *	Rod-like	20–50 × 80–220 nm	Water	DCM	W/O	Vortex mixer	3500 rpm n.d.	Scaffolds fabrication	[133]
P(LLA/CL) *	Spherical	50 nm	Water	DCM	O/W	Rotor-stator	20,450 rpm 3 min	Scaffolds fabrication	[134]
PLLA *	Spherical	30–70 nm	Water	DCM	W/O	Rotor-stator	12,000 rpm 1 min	Scaffolds fabrication	[135]
Alginate + PLLA *	Spherical	20–70 nm	Water	DCM	O/W	Rotor-stator	12,000 rpm 1.5 min	Scaffolds fabrication	[136]
Stearic acid + PLLA *	n.d.	n.d.	Water	DCM	O/W; W/O	Rotor-stator	17,000 rpm 1 min	PE stabilisation	[137]
PLLA *	n.d.	n.d.	Water	DCM	O/W; W/O	Rotor-stator	200–20,000 rpm 0.2–3 min	PE stabilisation	[26]
CTAB and PG + PLLA *	n.d.	0.2–1.2 µm	Water	DCM	O/W	Ultrasonic	250 W 5 min	PE stabilisation	[138]
Stearic acid; PLLA + Span 80 *	n.d.	n.d.	Water	DCM	O/W; W/O	Rotor-stator	10,000–20,000 rpm 0.5–4 min	PE stabilisation	[139]
PS *	Spherical	40 nm	Water	DCM	O/W	Vortex mixer	3200 rpm 1 min	PE stabilisation	[27]
Sodium oleate	Rod-like	23 × 70 nm	Water	Cy	W/O; O/W	Ultrasonic	300 W 6 cycles	PE stabilisation	[140]
Stearic acid	n.d.	30 nm	Water	n.d.	W/O	Magnetic stirrer	12,000 rpm n.d.	PE stabilisation	[141]
PMF	Spherical	30–70 nm	Water	Artemisia argyi oil	O/W	Rotor-stator	10,000 rpm 2 min	PE stabilisation	[142]
DBP	Rod-like	n.d.	Water	Hexanol	O/W	Ultrasonic	n.d.	Protocells fabrication	[143]
n.a.	Rod-like	50 nm	Water	Sunflower oil	O/W	Rotor-stator	11,000 rpm 6 min	PE stabilisation	[25]
n.a.	Rod-like	50 nm	Water	Sunflower oil	O/W	NETmix	200–500 Reynolds number 1–35 cycles	PE stabilisation	[144]
n.a.	Rod-like	50 nm	Water	Sunflower oil	O/W	NETmix	300–400 Reynolds number 5–17 cycles	Vitamin E-loaded PE	[114]
Sodium oleate	Rod-like	50 nm	Water	Sunflower oil	W/O	Rotor-stator	11,000 rpm 2 min	PE stabilisation	[48]

*—Dispersed in oil phase to modify the HAp surface during emulsification; CTAB—cetylmethylammonium bromide; Cy—cyclohexane; DBP—dibutyl phosphate; DCM—dichloromethane; DMF—dimethylformide; HAp—hydroxyapatite; n.a.—not applicable; n.d.—not defined; O/W—oil-in-water; PCL—poly(ε-caprolactone); PG—propyl gallate; PLLA—poly(L-lactic acid); P(LLA/CL—poly(L-lactide–co-ε-caprolactone); PMF—poly(melamine formaldehyde); PS—polystyrene; W/O—water-in-oil.

**Table 3:** Summary of characteristics, advantages and disadvantages of main emulsification devices used for PE production.

Homogenizer Type	Throughput	Efficiency	Droplet Size		Advantages	Disadvantages
			Control	Minimum		
High-shear	Batch	Low	Rotation speed Emulsification time	2 µm	Easy set-up Quick processes Low operating cost Small amounts of the liquids Different apparatus available	Particle disruption Temperature increase Broad droplet size Limited energy input
Ultrasonic	Batch	Low	Ultrasound frequency Amplitude Emulsification time	0.1 µm	Easy set-up Quick processes Small amounts of the liquids	Particle disruption Temperature increase Broad droplet size Probe degradation
High-pressure	Batch or continuous	High	Pressure value Number of homogenizing cycles	0.1 µm	Quick processes Narrow droplet size	Particle disruption Temperature increase High energy consumption Difficult to clean
Membrane	Batch or continuous	Very high	Membrane pore size Injection rate Agitation speed	0.3 µm	Particle integrity Temperature control Narrow droplet size Low energy consumption	Set-up Slow process Viscosity of the fluids
Microfluidizers	Continuous	High	Flow rate Microchannel geometry Number of cycles Phase viscosities	0.1 µm	Particle integrity Temperature control Droplet size control Narrow droplet size Multiple emulsion production Low energy consumption	Viscosity of the fluids Set-up Slow process
Static mixers	Continuous	High	Flow rate Number of cycles	0.3 µm	Particle integrity Mixing control Temperature control Droplet size control Low energy consumption	Viscosity of the fluids
